# Advances in Flow Chemistry for Organolithium-Based Synthesis: A Process Perspective

**DOI:** 10.3390/molecules31010105

**Published:** 2025-12-26

**Authors:** Feng Zhou, Yijun Zhou, Chuansong Duanmu, Yanxing Li, Jin Li, Haiqing Xu, Pan Wang, Kai Zhu

**Affiliations:** 1 National & Local Joint Engineering Research Center for Deep Utilization Technology of Rock-Salt Resource, Faculty of Chemical Engineering, Huaiyin Institute of Technology, Huai’an 223003, China; 2 China Construction Industrial & Energy Engineering Group Co., Ltd., Nanjing 210023, China

**Keywords:** organolithium, flow chemistry, microreactor, process intensification

## Abstract

While organolithium reactions hold great promise in synthetic chemistry, their high reactivity, strong exothermicity, and the instability of intermediates often limit their application, making the effective control of reaction processes difficult in traditional batch reactors. This review systematically summarizes the latest advances in utilizing flow chemistry technology to address process challenges related to organolithium reactions from 2014 to 2025. From a process perspective, we systematically discuss the literature cases regarding three key themes: the synthesis of organic compounds applied in the pharmaceutical field, the development of novel methods centered on effective process control (reaction temperature, residence time, phase state, multi-step reaction sequence, and safety), and fundamental process research on continuous flow organolithium reactions. Analysis shows that continuous flow systems provide a powerful platform for fully realizing the potential of organolithium chemistry by enhancing heat/mass transfer and precisely controlling reaction parameters. This review emphasizes how flow chemistry technology not only improves process safety and efficiency but also enables transformations and process scaling that are difficult or impossible in batch modes, thus providing a novel process intensification method for modern synthetic chemistry.

## 1. Introduction

Organometallic compounds play a key role in constructing organic molecular skeletons [1], and among commonly used organometallic compounds, organolithium compounds exhibit very significant reactivity. Since their discovery in the early 20th century, organolithium compounds have become an indispensable tool for constructing carbon–carbon (C-C) bonds and carbon–heteroatom (C-X) bonds [2]. Organolithium reactions such as deprotonation and lithium–halogen exchange play an irreplaceable role in synthetic chemistry. Despite the numerous advantages of organolithium reactions, their practical application is constrained by the incompatibility between traditional batch reactor systems and the inherent reaction characteristics of organolithium chemistry. The inherent high reactivity, strong exothermic properties, and difficult-to-suppress side reactions of organolithium reactions, as well as the high sensitivity of organolithium compounds to air and moisture, affect various aspects such as reaction process design, reaction process control, and process scale-up. For example, traditional organolithium reactions usually need to be carried out at low or even ultra-low temperatures to ensure the safety and controllability of the reaction, but this also leads to a high energy consumption and high cost. The inherent limitations of traditional batch reactor systems have prompted the widespread application of flow chemistry, a novel process intensification technology, in organolithium reactions [3,4,5,6]. Through efficient mixing, enhanced transport, and precise process parameter control at the micro-scale, the target product selectivity and process safety of organolithium reactions have been significantly improved. The key to the effectiveness of flow chemistry in overcoming the challenges of the practical applications of organolithium chemistry lies in its ability to provide a fundamentally safer, more efficient, precisely controlled, and easily scalable synthetic platform for organolithium reactions through its micro-scale characteristics such as miniaturization and high specific surface area. It not only addresses the inherent defects of batch reactors (including insufficient thermal management, limited mixing efficiency, poor intermediate stability control, and difficulty in scale-up) but also creates complex and extreme reaction conditions that were previously impossible to achieve in batch reactors [7,8,9,10]. Flow chemistry provides effective support for unleashing the potential of organolithium reactions in the synthesis of high-value molecules (such as key intermediates for novel drugs) [11,12].

A recent review by Mauro Spennacchio et al. highlights the continuous flow generation of highly reactive organolithium reagents, summarizing advances from the past four years, including applications in synthesizing biologically relevant compounds [13]. Aiichiro Nagaki extensively documented the capabilities of flow microreactors in advancing organolithium chemistry across their review [14]. The review emphasized how precise temporal control in flow systems facilitates the engagement of unstable intermediates, enabling the synthesis and subsequent reactions of functional organolithiums—including functional alkyllithiums, functional benzyllithiums and aryllithiums bearing cyano, alkoxycarbonyl, nitro, and ketone carbonyl groups. This approach supports direct reaction integration, achieving transformations that are impractical in traditional macro batch reactors. Additionally, Aiichiro Nagaki and colleagues detailed spatial integration methodologies for multi-step organolithium-mediated reactions, introducing both linear integration and convergent integration [15]. These strategies encompass reactions such as halogen–lithium exchange, deprotonation, carbolithiation, polymerization, and coupling reactions, highlighting the effectiveness, robustness, and adaptability of flow microreactors in complex synthetic pathways. Kengo Inoue et al. addressed trapping methods of short-lived organolithium species, while also discussing reaction types involving organolithiums and summarizing synthetic methodologies utilizing these reactions [16]. Johannes H. Harenberg et al. reviewed advances in the application of flow chemistry to organolithium reactions, covering the generation of organolithium reagents, the preparation of acyllithiums and lithium carbenoids, and demonstrating the technology’s advantages in overcoming the limitations of traditional batch processes [17]. The review by Mark Power et al. focused on deprotonation reactions using organolithium bases in flow chemistry, evaluating the performance of common organolithium bases such as *n*-butyllithium (*n*-BuLi) and lithium diisopropylamide (LDA) in continuous flow systems [18]. Through case studies, the authors illustrated how flow chemistry significantly enhances reaction yield, efficiency, and safety.

While many reviews have explored the application of flow chemistry technology in organolithium reactions, they have primarily focused on a chemical perspective. This review aims to shift this focus, offering a systematic analysis from a process engineering standpoint to elucidate how this technology can achieve process intensification. This approach not only highlights the crucial role of continuous flow systems in overcoming the long-standing limitations of conventional batch reactions but also points to their emerging applications in modern organic synthesis. Subsequent sections will delve into specific case studies to provide valuable insights for advancing the practical application of organolithium chemistry.

## 2. Flow Chemistry Toward Organolithium-Based Synthesis in the Pharmaceutical Field

**Figure 1:**
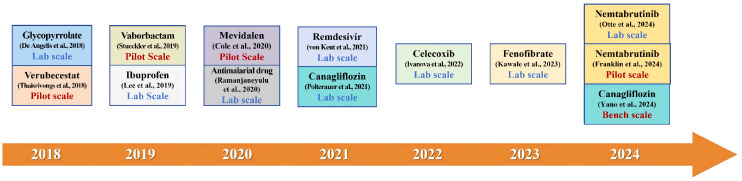
Flow chemistry toward organolithium-based synthesis in the pharmaceutical field (2018–2024) [19,20,21,22,23,24,25,26,27,28,29,30,32]. (Note: Each distinct colored block corresponds to a specific compound, with identical colored blocks representing the same compound).

Flow chemistry technology has been successfully implemented to construct critical steps in the production of numerous significant compounds, demonstrating enhanced efficiency and control over traditional batch methods. In this section, we examine the key role of flow chemistry in facilitating organolithium reactions for the synthesis of high-value compounds in the pharmaceutical field. Its broad adoption in the preparation of commercial drugs, approved active pharmaceutical ingredients, and investigational drug candidates is well documented in the literature [19,20,21,22,23,24,25,26,27,28,29,30,31,32]. Figure 1 presents a timeline (2018–2024) of flow chemistry applications in critical organolithium-based synthesis in the pharmaceutical field, encompassing both lab-scale and pilot-scale implementations. The diverse range of organolithium reactions highlighted in these applications displays the versatility and robustness of flow chemistry in following fast and complex synthetic pathways.

**Figure 2:**
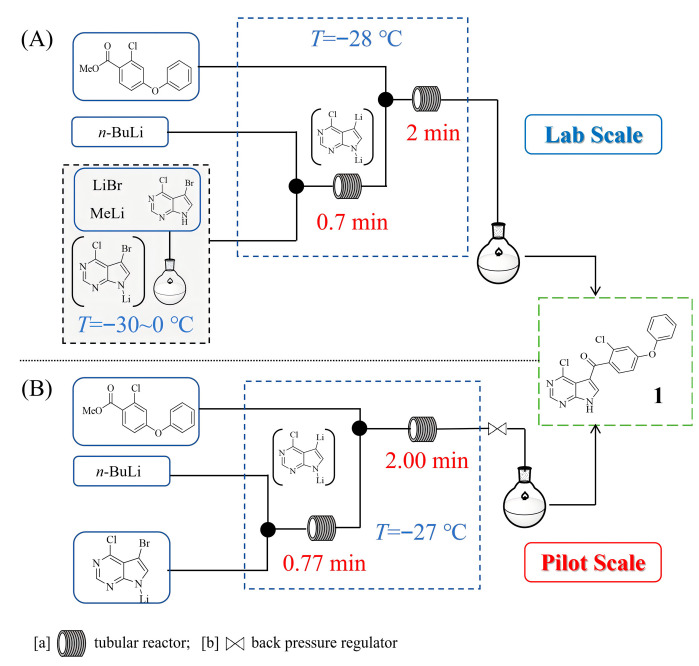
Continuous flow organolithium-mediated synthesis of a key ketone intermediate **1** to nemtabrutinib. (**A**) Designed process for lab scale. (**B**) Designed process for pilot scale.

Nemtabrutinib is a novel reversible Bruton’s tyrosine kinase (BTK) inhibitor developed for treating B-cell cancers. Douglas A. L. Otte et al. [19] developed a continuous flow method for the synthesis of a ketone intermediate for nemtabrutinib, which highlights the transition from a batch process to a hybrid batch-flow system. Initially, the batch method relied on simultaneous deprotonation and lithiation with *n*-BuLi at cryogenic temperatures (−60 °C), resulting in a modest 61% yield of **1** and the significant formation of dehalogenated impurities. By implementing a sequential deprotonation step with methyllithium in batch reactors followed by lithiation with *n*-BuLi in a flow reactor (Figure 2A), the process achieved a 14% assay yield improvement of **1** and allowed operation at a higher temperature of −28 °C, reducing energy demands and enhancing robustness. The incorporation of lithium bromide as an additive maintained homogeneity in the plug flow reactor, enabling a 90% reduction in residence time compared with initial continuously stirred tank reactor designs, and demonstrated the flow system’s superiority in controlling reactive intermediates. Building on these lab-scale advancements, the process was successfully scaled to pilot-level production, as described by Robert D. Franklin et al. [20], where a tubular flow reactor was employed for the multi-kilogram synthesis of the same ketone intermediate (Figure 2B). This campaign focused on practical operational aspects, such as equipment design with static mixers and residence time units, precise flow rate control using gear pumps and mass flow controllers, and temperature maintenance around −30 °C to manage exotherms. Over five batches, the process delivered a consistent performance with an average isolated yield of ~65% and purity of 99.9% by weight, while off-target experiments revealed that *n*-BuLi stoichiometry had the greatest impact on reaction yield.

**Figure 3:**
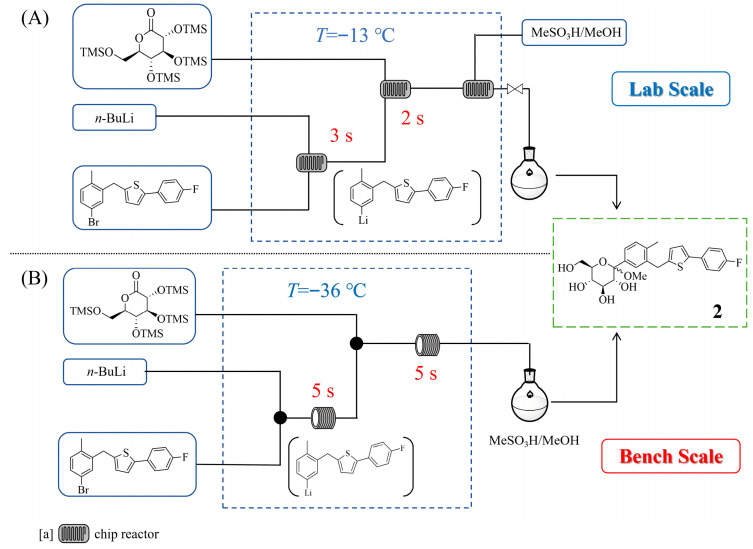
Continuous flow process for the organolithium-mediated synthesis of a key intermediate **2** to canagliflozin. (**A**) Designed process for lab scale. (**B**) Designed process for bench scale.

Canagliflozin, a sodium–glucose cotransporter 2 (SGLT-2) inhibitor, is widely used for treating type 2 diabetes mellitus. Dominik Polterauer et al. [21] developed a telescoped batch-flow hybrid process for the synthesis of the key intermediate of canagliflozin. This approach involved a three-step sequence—lithiation, C-arylation, and methoxylation—within a microstructured flow reactor (Figure 3A), enabling precise control over exothermic reactions and mixing sensitivity. By employing a design of experiments (DoE) strategy, they optimized parameters such as temperature (−13 °C), residence time (3 s for the lithium–halogen exchange and 2 s for the C-arylation), and reagent stoichiometry (1.2 equiv *n*-BuLi and lactone) to achieve a 76% isolated yield of intermediate **2** and a high throughput of 26.8 g/h. This method eliminated the need for cryogenic conditions (−13 °C in flow versus −70~−80 °C in batch) and enhanced sustainability through improved space–time yields. In addition, Gemba Yano et al. [22] further advanced the continuous flow process by addressing scalability and clogging issues using plug flow reactor (PFR) and coflore agitated cell reactor (ACR) systems for the synthesis of intermediate **2**. Their study optimized the Br/Li exchange and C-arylation steps, demonstrating that 1.1 equiv of *n*-BuLi and short residence times (15 s for the lithium–halogen exchange and 5 s for the C-arylation) could achieve up to 88% yield of **2** under a controlled temperature (−20 °C). The PFR system allowed scale-up to a bench level (Figure 3B) with a throughput of 3.4 kg/h for the desired product **2**, while the ACR setup provided greater robustness against insoluble by-products but had a poorer heat removal efficiency.

**Figure 4:**
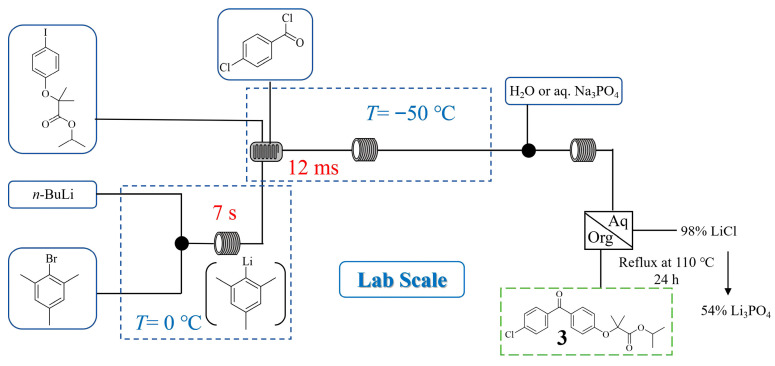
Sequential flow synthesis of fenofibrate followed by product isolation and in-line recovery.

Fenofibrate is a widely prescribed drug for treating cardiovascular diseases. Sanket A. Kawale et al. [23] developed a compact, monolithic metal microreactor fabricated via 3D printing to conduct the rapid lithium–halogen exchange and subsequent coupling reaction for fenofibrate production (Figure 4). This flow chemistry approach successfully controlled the highly unstable aryllithium intermediate at a large scale using a monolithic eight numbered-up microreactor, achieving a throughput of 1.18 g/min with 76% yield of fenofibrate **3**. Besides the novel design and manufacturing of the microreactor structure, a key innovation involved the in-line recovery of lithium resources. Specifically, the lithium consumed in the continuous flow process was recovered in high yield, as LiCl (98% yield) when quenched with water, or as Li_3_PO_4_ (54% yield) when quenched with aqueous Na_3_PO_4_.

**Figure 5:**
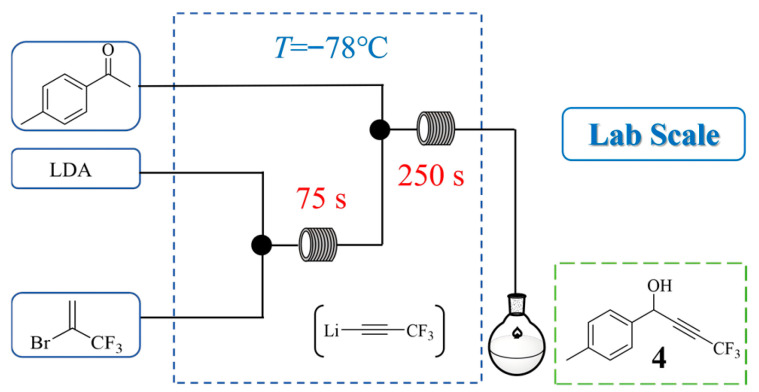
Continuous flow process for the organolithium-mediated synthesis of the intermediate **4** to Celecoxib.

Celecoxib is a widely prescribed anti-inflammatory COX-2 inhibitor used for treating inflammation and pain in various types of arthritis. Maria Ivanova et al. [24] developed a continuous flow route to produce Celecoxib starting from 2-bromo-3,3,3-trifluoropropene. The process involves the generation of a highly reactive trifluoropropynyl lithium intermediate under cryogenic conditions (−78 °C), which is immediately trapped with p-tolualdehyde to form a key alcohol intermediate **4** (Figure 5). Within a total residence time of only 5 min 25 s, the intermediate was obtained in 62% yield and was then subsequently oxidized and condensed with 4-sulfamidophenylhydrazine to yield Celecoxib. The flow system enabled the safe handling of unstable organolithium species and achieved a total residence time of approximately one hour over three steps, providing an overall Celecoxib yield of 50%.

**Figure 6:**
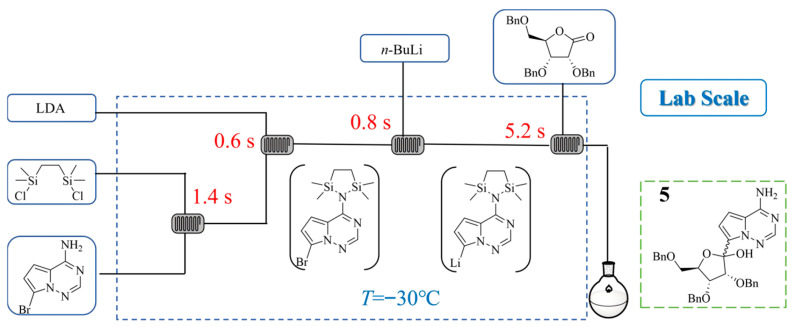
Continuous flow process for the organolithium-mediated synthesis of a key intermediate **5** to remdesivir.

Remdesivir is an antiviral drug approved for treating COVID-19. In its synthesis, the organolithium-mediated C-glycosylation step was a key bottleneck, requiring cryogenic conditions and prolonged addition times in batch processing. Timo von Keutz et al. [25] developed a continuous flow approach using a microreactor to perform lithium–halogen exchange and subsequent nucleophilic addition with a ribonolactone (Figure 6). This flow chemistry method operated at a significantly higher temperature (−30 °C) with a very short total residence time of 8 s, achieving a 60% yield of the glycosylated intermediate **5**. A stable process was demonstrated over 2 h in a scalable flow reactor system, reaching an exceptionally high space–time yield of 10.4 kg·L^−1^·h^−1^.

**Figure 7:**
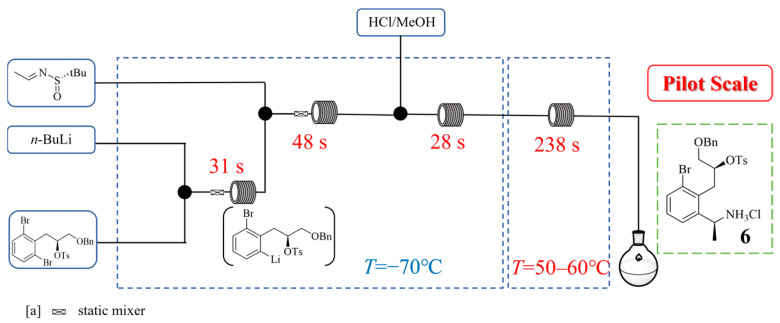
Continuous flow process for the organolithium-mediated synthesis of a key hydrochloride intermediate **6** to mevidalen.

Mevidalen is a dopamine D1 receptor positive allosteric modulator and its 4-hydroxybenzoic acid cocrystal is developed as a potential treatment for treating neurodegenerative disorders. In its synthesis, Kevin P. Cole et al. [26] developed a scalable continuous flow process that involves a cryogenic lithium–halogen exchange and addition to a chiral imine (Figure 7), which was applied to the large-scale synthesis of 185 kg of a key tetrahydroisoquinoline intermediate for mevidalen. This work successfully redesigned the synthetic route to avoid the expensive 2-bromo-D-phenylalanine starting material, utilizing commercially available 1,3-dibromobenzene and (S)-benzyl glycidyl ether instead. The critical innovation involved a flow-controlled lithium–halogen exchange and subsequent addition to the imine at −70 °C under precise residence time control to handle the highly unstable aryllithium intermediate, after which the mixture was subsequently treated with aqueous HCl in methanol and heated in continuous flow to remove the chiral auxiliary. The continuous flow system using plug flow reactors with static mixing elements enabled exact control over the highly exothermic reaction and afforded the hydrochloride intermediate **6**. Subsequent batch operations of cyclization, aqueous work-up, and heminapthalene-1,5-disulfonic acid (NDSA) were adopted to convert **6** to NDSA salt. The pilot plant campaign delivered 405 kg of NDSA salt with 66% overall yield, achieving high purity levels of 95.3–97.1% (HPLC area%).

**Figure 8:**
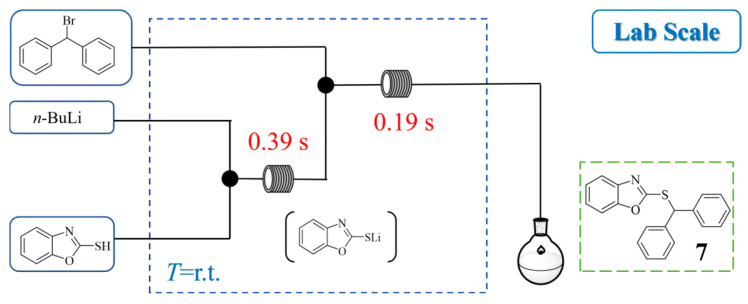
Continuous flow process for the synthesis of the antimalarial drug of 2-(benzhydrylthio) benzo[d]oxazole **7**.

2-(Benzhydrylthio)benzo[d]oxazole has attracted considerable interest due to its demonstrated antimalarial activity in vitro and in vivo against drug-resistant malaria. Bandaru T. Ramanjaneyulu et al. [27] developed an ultrafast continuous organolithium-mediated process for synthesizing 2-(benzhydrylthio)benzo[d]oxazole (Figure 8). The method involves generating a highly unstable lithium thiolate intermediate in situ from benzo[d]oxazole-2-thiol and *n*-BuLi, which subsequently reacts with benzhydryl bromide in a capillary microreactor at room temperature. This flow system drastically reduced the reaction time from 10 min in batch to just 580 ms, achieving a 75% yield of **7** while effectively controlling the reactive intermediate. The process demonstrated a high throughput, producing approximately 4 g of the target compound **7** in 13.3 min at a high flow rate of 7.6 mL/min.

**Figure 9:**
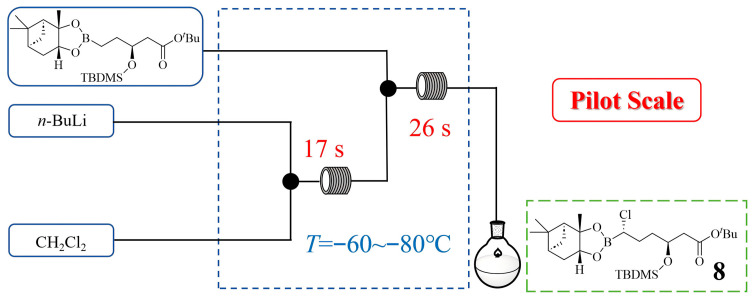
Continuous flow process for the Matteson reaction to synthesize the key intermediate of α-chloroboronic ester **8**.

Vaborbactam, a cyclic boronic acid *β*-lactamase inhibitor, can be used in combination with meropenem for treating complicated urinary tract infections and pyelonephritis. In its synthesis, the Matteson reaction—a critical step involving the generation and reaction of unstable (dichloromethyl)lithium—posed significant challenges in batch mode due to stringent cryogenic requirements. Clemens Stueckler et al. [28] successfully implemented a continuous flow process to address this issue, enabling the precise control of the reactive organolithium intermediate at temperatures as low as −60~−80 °C with residence time controlled to 43 s in tubular reactors (Figure 9). Guided by a detailed understanding of the optimal reaction conditions, a production-scale flow reactor was assembled and operated to yield several hundred kilograms of intermediate **8** in high purity (>98%) and yield (91%) during the registration campaign. The Matteson reaction transitioned from an unscalable lab batch to continuous manufacturing, proving continuous flow feasibility for large-scale pharmaceutical production.

**Figure 10:**
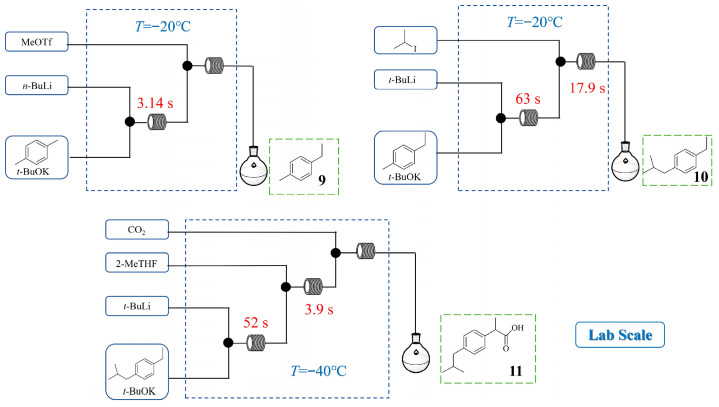
Continuous flow approach for the three-step synthesis of ibuprofen from *p*-xylene.

Ibuprofen is a widely used analgesic, antipyretic, and nonsteroidal anti-inflammatory drug for pain and fever relief. Hyune-Jea Lee et al. [29] developed a three-step continuous flow synthesis method starting from inexpensive p-xylene (Figure 10), utilizing sequential chemoselective C-H metalations mediated by an in situ generated LICKOR-type superbase. The continuous flow platform enabled the precise control and optimization of reaction conditions, effectively suppressing side reactions and achieving high yields of the target products (95% yield of **9** and **10** for the first two steps). The final step involved a biphasic flow reaction between a lithiated intermediate and CO_2_ and the three-step synthesis produced ibuprofen **11** at the gram-scale (2.3 g in 10 min) with a 57% isolated yield. By controlling reactive organolithiums and enabling chemoselective metallation with superbases, flow chemistry provides an effective strategy for synthesizing diverse bioactive molecules.

**Figure 11:**
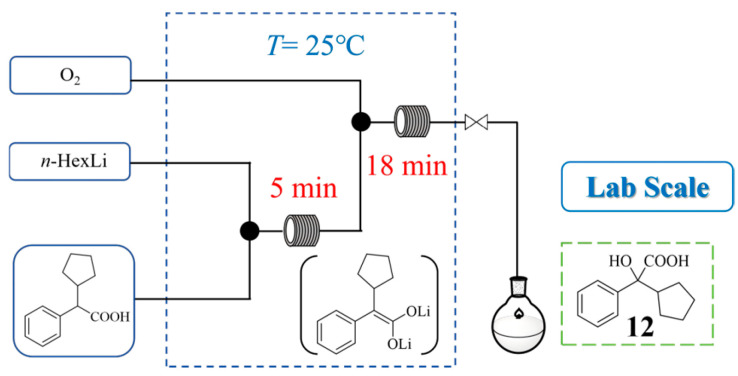
Continuous flow process for sequential *α*-lithiation using HexLi and aerobic oxidation.

Glycopyrronium bromide is an anticholinergic drug, which can be used to treat sialorrhea and hyperhidrosis. In the continuous flow synthesis of its key intermediate, cyclopentylmandelic acid (CPMA), Sonia De Angelis et al. [30] developed a sequential α-lithiation and aerobic oxidation process using *n*-hexyllithium (*n*-HexLi) as a safe and efficient organolithium base (Figure 11). The flow reactor enabled precise control over reactive organolithium intermediates and the direct use of pure oxygen, mitigating the safety risks associated with batch methods. Following a comprehensive investigation of process parameters, a telescoped continuous flow process was established with the following optimized conditions: residence times of 5 min for α-lithiation and 18 min for aerobic oxidation, reaction temperature 25 °C, and 0.6 equiv. of O_2_. This process afforded CPMA **12** in a 65% NMR yield, with an isolated yield of 50% after recrystallization, thereby rivaling conventional Grignard-based approaches.

**Figure 12:**
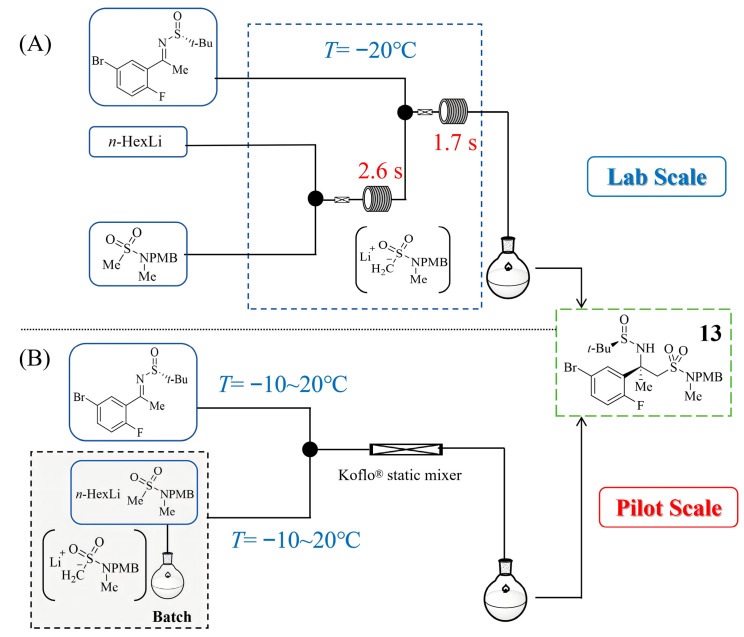
Continuous flow process towards the synthesis of the key intermediate **13** for verubecestat. (**A**) Designed process for lab scale. (**B**) Designed process for pilot scale.

Verubecestat (MK-8931) is an inhibitor of the aspartic protease beta-secretase 1, which has been evaluated in Alzheimer’s disease clinical trials. David A. Thaisrivongs et al. [31] demonstrated that a Mannich-type addition of an organolithium species to a chiral sulfinyl ketimine **13**—a key step in the synthesis of verubecestat—suffered from competitive proton transfer between the desired and undesired reactions under batch conditions, resulting in moderate yields and requiring cryogenic temperatures. By transitioning the synthesis to a continuous flow system, the authors significantly enhanced mixing efficiency, which suppressed unexpected side reactions and allowed the reaction to proceed effectively at non-cryogenic temperatures (Figure 12A). This approach not only improved the assay yield of **13** from 73% in batch to 87–91% but also demonstrated the critical role of micromixing in controlling highly reactive organolithium intermediates and the clogging risks on the experiments at kilogram scale. Building on these findings, the same group later reported the pilot-scale development of this transformation (Figure 12B), addressing challenges such as reactor fouling and intermediate instability [32]. They introduced N,N′-dimethylpropyleneurea as a solubility-enhancing additive for the lithiated species and implemented an advanced process analytical technology for real-time monitoring. Using pilot plant-scale static mixers, the team achieved consistent operation over several hours under an optimized continuous process, producing >100 kg of product in a single batch with consistent assay yields of 88~89%. This work indicated the scalability and robustness of flow chemistry for organolithium-mediated reactions under an optimized continuous process.

These studies illustrate how flow chemistry can overcome the fundamental limitations of batch processing in organolithium-based synthesis, enabling safer, more efficient, and scalable manufacturing strategies for the synthesis of complex organic compounds in the pharmaceutical field.

## 3. Flow Chemistry in Novel Organolithium-Based Methodologies

**Figure 13:**
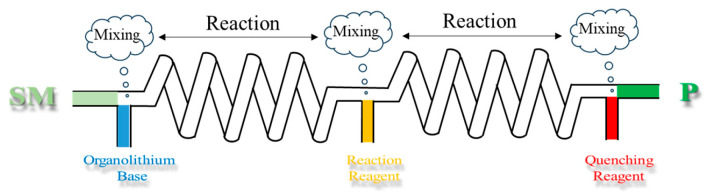
Universal flow process diagram for organolithium-based reactions (SM—starting material, P—product).

The integration of flow chemistry into organolithium reactions has effectively improved synthetic methodology by enabling precise control over reaction behaviors that are difficult to manage in traditional batch systems. The foundation of these advancements lies in the diverse characteristics of organolithium species and the specifically designed continuous flow system. Organolithium species can be generated through various methods such as deprotonative lithiation and halogen–lithium exchange. During the organolithium reactions, organolithium bases serve as powerful tools to generate key carbanion intermediates for various bond-forming processes. The choice of organolithium base directly influences optimal reaction conditions, necessitating a careful balance of pKa, nucleophilicity, and steric effects [18]. Upon determining the organolithium bases involved in the process, the next step involves constructing a continuous flow system based on the reaction characteristic. Figure 13 depicts a generalized continuous flow process for organolithium-based transformations. This diagram shows how reaction steps such as lithiation, electrophilic trapping, and quenching are integrated into a sequential continuous flow pathway. Organolithium reactions often involve multiple steps in practical applications and such processes can be categorized as either linearly integrated or convergently integrated [15]. In linear integration, two components react to form a short-lived intermediate, which subsequently generates other short-lived intermediates upon the addition of further reactants. This linear sequence can be repeated within a single flow process to yield the target molecule. In convergent integration, multiple distinct short-lived intermediates are generated separately and combined to construct the target molecule. Additionally, complex molecule synthesis may simultaneously employ both linear and convergent integration strategies. This section systematically examines five critical dimensions underpinning the development of robust and innovative continuous flow synthetic strategies based on organolithium reactions: reaction temperature, residence time, phase regime, synthetic steps, and process safety.

### 3.1. Reaction Temperature (T)

Traditional batch processes face significant challenges in handling highly reactive, exothermic organolithium reactions due to inherent limitations in mass and heat transfer. To suppress side reactions from poor mixing or localized overheating, such reactions often require ultra-low temperatures, leading to high energy consumption and limited scalability. In contrast, flow chemistry utilizes microreactors with high surface-area-to-volume ratios to achieve rapid mixing and efficient heat transfer, enabling precise control over reaction conditions and minimizing side reactions. This allows organolithium reactions to be conducted safely at significantly higher temperatures while maintaining or improving selectivity and yield.

**Figure 14:**
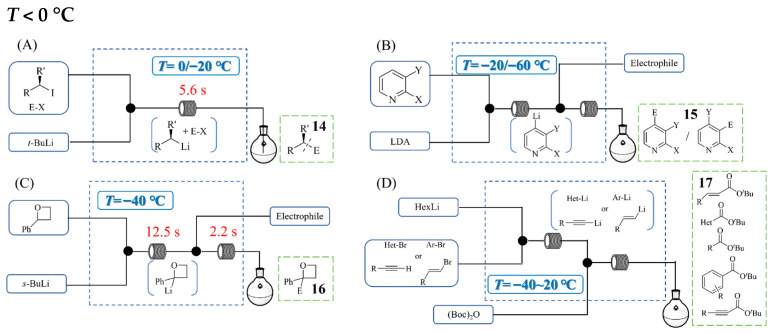
Continuous flow process for organolithium reactions at <0 °C. (**A**) The in situ quenching of chiral secondary alkylithiums. (**B**) The divergent lithiation of 2,3-dihalopyridines. (**C**) The deprotonation of 2-phenyloxetane with *s*-BuLi followed by reaction with an electrophile. (**D**) Direct tert-butoxycarbonylation.

The enantioselective synthesis of small molecules is attracting growing interest in pharmaceutical/agrochemical research, as preparing enantioenriched organometallics directly produces enantiopure compounds by electrophile quenching. Alexander Kremsmair et al. [33] developed a practical in situ quench procedure that generates chiral secondary alkyllithiums in the presence of various electrophiles, which enabled the preparation of a broad range of enantiomerically enriched ketones, alcohols, amides, sulfides, and boronic acid esters with typical enantiomeric excesses of 90–98%. By leveraging enhanced mixing and heat transfer in a continuous flow system (Figure 14A), the authors successfully performed the in situ quench reactions under continuous flow conditions at elevated temperatures between −20 °C and 0 °C (−78 °C/−40 °C in batch), achieving comparable yields and a high enantiomeric excess for various functionalized products **14**. This approach not only improved operational practicality by reducing cooling demands but also enabled a 40-fold scale-up without further optimization. The reaction with 2-halogen-3-bromo and 2-halogen-3-iodopyridine (batch, LDA base) required cryogenic temperatures (−70 °C) to minimize side products and the products resulting from the halogen dance were highly base-sensitive at high temperatures. Thibaud Brégent et al. [34] reported the selective lithiation of 2,3-dihalopyridines under continuous flow conditions at −20/−60 °C to obtain products **15** (Figure 14B). The halogen dance was performed in continuous flow conditions on 2-chloro-3-bromopyridine by selectively trapping a (pyridin-4-yl)lithium species; this approach was then extended to fluoroiodopyridines using diverse electrophiles, affording 28 examples in 42–97% yields. In addition, this continuous flow process can be readily scaled up with a space–time yield of 4.2 kg·h^−1^·L^−1^. Oxetane rings serve as important building blocks in pharmaceutical and natural product synthesis. Leonardo Degennaro et al. [35] demonstrated that a continuous flow system enables the generation and electrophilic trapping of highly unstable organolithium intermediates for the synthesis of oxetane derivatives **16** at substantially elevated temperatures (−40 °C) compared with batch processing (−78 °C) (Figure 14C). The flow process—through the precise control of residence time (optimized at 12.5 s) and efficient heat management—achieved moderate to good yields for the reactions with diverse electrophiles (e.g., 85% yield for 2-phenyl-2-trimethylsilyl oxetane) at −40 °C. Leonardo Degennaro et al. [36] also developed a direct and sustainable continuous flow process for the synthesis of tertiary butyl esters **17** from organolithiums and di-*tert*-butyldicarbonate (Boc)_2_O (Figure 14D). In batch mode, this chemical transformation required heavy cryogenic conditions to suppress unexpected reactions. By transferring the reaction to the flow mode, the authors achieved precise control over residence time and temperature, enabling the efficient C-*tert*-butoxycarbonylation of highly reactive organolithiums at significantly higher temperatures (−40~20 °C) for different substrates. After optimization, the continuous flow process afforded the desired esters **17** in high yields (e.g., up to 90% yield for *tert*-butyl 4-chlorobenzoate) while minimizing side product formation. In comparison with the batch process, the developed flow process for the synthesis of tertiary butyl esters offered a superior efficiency, broader versatility, and greater sustainability.

**Figure 15:**
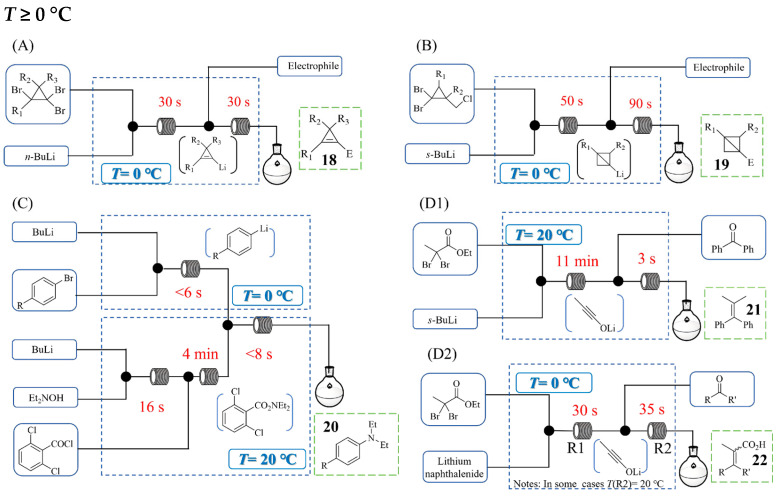
Continuous flow process for organolithium reactions at ≥0 °C. (**A**) The generation and use of cyclopropenyllithium. (**B**) The generation and use of bicyclo[1.1.0]butyllithium. (**C**) Three-step-integrated electrophilic aminations. (**D1**,**D2**) The synthesis and reactions of ynolates ((**D1**) uses a stop-flow method, and (**D2**) employs reductive lithiation for ynolate generation).

Compared with controlling sub-zero temperatures (<0 °C), managing temperatures at or above 0 °C is notably more straightforward and manageable. Francesco Soddu et al. developed a continuous flow process (Figure 15A) for generating and functionalizing cyclopropenyllithium that significantly elevates reaction temperatures compared with traditional batch methods [37]. Their flow system successfully operates at 0 °C using *n*-BuLi, completely avoiding the cryogenic conditions and laborious cool–warm–cool temperature cycles required in batch protocols. Comparing productivity, space–time yields, and process time for several cyclopropene syntheses at identical scales (batch vs. flow), the authors found the flow method consistently outperformed batch processing—with up to 400% higher productivity, 270-fold higher space–time yields, and a 57% shorter process time. The flow approach enables efficient reactions with diverse electrophiles for the synthesis of cyclopropene derivative **18** under significantly milder conditions. Elena Graziano et al. [38] developed a continuous flow process for the generation and electrophilic trapping of highly reactive bicyclo[1.1.0]butyllithium (Figure 15B), in which the reaction temperature was significantly elevated to 0 °C compared with traditional batch methods (−78 °C). This flow process used *s*-BuLi, a single organolithium reagent that is less hazardous and more practical than MeLi and *t*-BuLi, and 2-methyltetrahydrofuran as a greener alternative for bicyclobutyllithium generation. The methodology was applied to a broad electrophile scope, such as aldehydes, ketones, Weinreb amides, and imines, yielding diverse bicyclo[1.1.0]butyl derivatives **19** in high yields. Heejin Kim et al. [39] developed a catalyst-free continuous flow process for the electrophilic amination of functionalized aryllithiums. The authors first designed and optimized a new aminating reagent, *O*-(2,6-dichlorobenzoyl)-*N,N*-diethylhydroxylamine, which effectively suppresses undesired side reactions to the carbonyl carbon atom. By employing a continuous flow system, the team successfully conducted the amination of various functionalized aryllithiums at 0 °C, a significantly higher temperature than the cryogenic conditions typically required in batch mode, enabling the synthesis of diverse aniline derivatives in high yields with excellent functional group tolerance. The process was further integrated into a sequential one-flow system (Figure 15C) to give the desired products **20** (63~80% yields), generating the aryllithium and aminating reagent in separate streams before combining them to achieve the complete transformation within 5 min. In the work of Toshiya Yoshiiwa and colleagues [40], the synthesis of ynolates, which traditionally requires strict low-temperature conditions (e.g., −78 °C) in batch systems to suppress side reactions, was successfully performed at ambient temperature using a flow microreactor system. Figure 15(D1) depicts a typical continuous flow process for the synthesis of ynolates and their subsequent reaction to form (2-methylprop-1-ene-1,1-diyl)dibenzene **21**. By employing a stop-flow method with a stainless-steel static micromixer, ynolates were generated within 11 min via Li–Br exchange with s-BuLi, contrasting sharply with the hour-long batch process. In a complementary approach, the same group utilized reductive lithiation with lithium naphthalenide in a continuous flow system to generate ynolates at 0 °C or room temperature within 1 min [41]. Figure 15(D2) shows the synthesis of tetrasubstituted alkenes **22** in good yields of 60~80% via ynolates using the continuous flow system. This method circumvented issues that had plagued earlier flow attempts using alkyllithium reagents, which sought to generate ynolate species via lithium naphthalenide-mediated reductive lithiation instead of Li/Br exchange. The system enabled the efficient olefination of carbonyl compounds with yields comparable to batch methods but under milder and faster conditions.

These cases demonstrate how flow microreactors provide precise thermal management, allowing organolithium reactions to proceed at elevated temperatures without compromising yield or selectivity, thereby enhancing both practicality and scalability in synthetic applications.

### 3.2. Residence Time (t)

Flow chemistry, leveraging a micro-scale channel structure, achieves the millisecond-level rapid and uniform mixing of reaction materials along with highly efficient mass/heat transfer, enabling precise control over residence time from milliseconds to seconds. The precise control of residence time, a core advantage of this technology, ensures that highly reactive organolithium intermediates react with target reagents immediately after formation, thereby suppressing side reactions such as decomposition. As a result, this technology enables highly selective transformations that are unachievable under conventional batch conditions.

**Figure 16:**
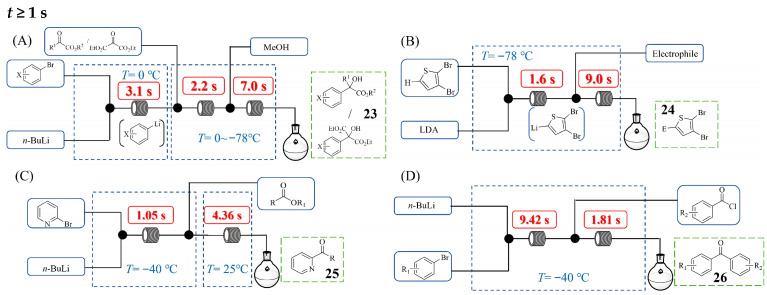
Continuous flow process for organolithium reactions with minimum residence time ≥ 1 s. (**A**) The efficient and selective transformations of α-keto esters into α-arylated α-hydroxy esters. (**B**) The generation and trapping of thienyllithiums. (**C**) The synthesis of 2-pyridyl ketone library from 2-lithiopyridine and esters. (**D**) The reaction of lithiated aromatic bromides with various acid chlorides.

Figure 16 illustrates the examples of continuous flow processes designed for organolithium reactions requiring a minimum residence time of 1 s or longer. Mohmmad S. Qenawy et al. [42] developed a continuous flow system enabling the precise control of residence time and temperature for the reaction of functionalized aryllithium reagents with α-keto esters into α-arylated α-hydroxy esters **23** (Figure 16A), leading to significantly improved yields and selectivity. This three-step flow synthesis involved a lithium–halogen exchange to generate the aryllithium species, its subsequent reaction with an α-keto ester, and a final quenching step, with residence times precisely controlled at 3.1 s, 2.2 s, and 7.0 s, respectively. The study on mixing efficiency by varying flow rates and inner diameters of micromixers demonstrated that optimal mixing conditions are crucial for enhancing reaction yield and selectivity. This flow approach effectively suppressed side reactions (e.g., competitive over-addition and decomposition pathways) that plague batch processes, enabling the successful handling of even highly challenging substrates, from sterically hindered α-keto esters to those bearing multiple reactive ester groups. In a study by Kentaro Okano et al. [43], a flow microreactor was employed to generate and trap transient thienyllithium species derived from 2,3- or 2,5-dibromothiophene via deprotonation with LDA. By precisely adjusting the residence time in the microtube reactor, the authors achieved the selective interception of the kinetic lithiated intermediate before it underwent halogen dance side reactions. Using the continuous flow system shown in Figure 16B with benzaldehyde as the electrophile, the desired product **24** was (4,5-dibromothiophen-2-yl)(phenyl)methanol and could be obtained with a 66% yield with only 3% isomerization by-product under the optimized conditions (1.6 s at −78 °C). In addition, a longer residence time (3.1 s at −78 °C) led to increased isomerization, affording the desired product in only 49% yield alongside 20% of the isomerization by-product. The established flow system was broadly applicable to diverse carbonyl compounds, giving good yields, and the contrasting results for the conversion of 2,3- and 2,5-dibromothiophene were explained by reaction pathway analysis. Maolin Sun et al. [44] developed a rapid and practical method for constructing a library of 2-pyridyl ketones **25** via bromine–lithium exchange to generate 2-lithiopyridine and a subsequent reaction with commercially available esters (Figure 16C). Utilizing a flow setup comprising T-mixers and microtube reactors, the authors achieved precise control over reaction parameters, with the critical Br–Li exchange step optimized to occur within 1.05 s at −40 °C, followed by immediate nucleophilic addition to esters in just 4.36 s at 25 °C. This ultrafast, combined residence time of under 5.41 s was essential for maintaining the stability of the highly reactive 2-lithiopyridine intermediate and preventing its decomposition, which plagues traditional batch methods. The precisely controlled flow process enabled the efficient synthesis of a diverse 20-membered ketones library in moderate to good yields (e.g., 80% yield for (4-chlorophenyl)(pyridin-2-yl)methanone), showing excellent functional group tolerance. Furthermore, the methodology was successfully applied to the synthesis of a key intermediate for the TGF-*β* receptor I inhibitor LY580276, achieving a 67% yield. Soo-Yeon Moon et al. [45] developed a continuous flow method for the direct synthesis of ketones **26** from acid chlorides and organolithiums (Figure 16D), achieving precise control over reaction parameters to suppress the over-addition side reactions that plague traditional batch methods. Key to their success was effective mixing and the reduction in the total residence time to just 11.2 s at −40 °C, which allowed the generated ketone to be rapidly transported away from the reaction zone before it could react further with organolithium reagents. This flow approach enabled the chemoselective synthesis of various functionalized ketones and the successful scale-up of the process to a 5 mmol scale afforded the product 2,2-dimethyl-1-(thiophen-3-yl)propan-1-one with an 85% yield, demonstrating its practicality for larger-scale operations.

**Figure 17:**
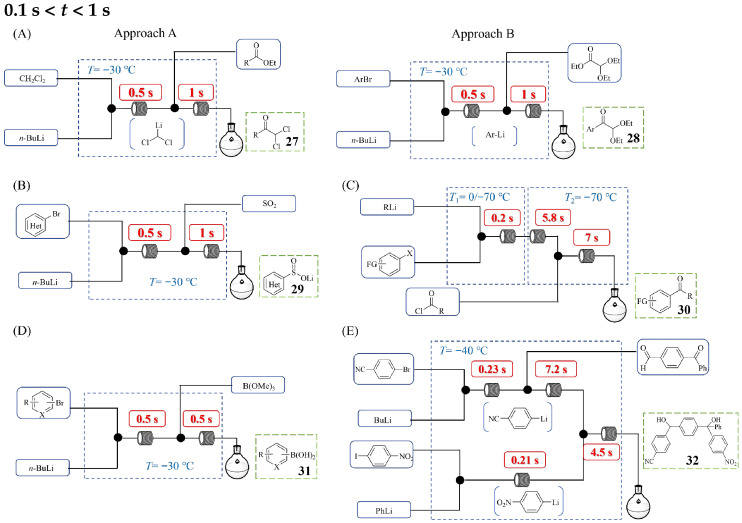
Continuous flow process for organolithium reactions with minimum residence time between 0.1 s and 1 s. (**A**) The synthesis of functionalized glyoxal derivatives. (**B**) The synthesis of heteroaryl sulfinates. (**C**) General process for reactions of functional acid halides with functional organolithiums. (**D**) The synthesis of boronic acids within 1 s. (**E**) Integrated continuous flow system for the chemoselective three-component coupling.

Figure 17 depicts the continuous flow system for organolithium reactions, in which the step with the minimum residence time proceeds within a narrow, sub-second window (between 0.1 and 1 s). Fabio Lima et al. [46] demonstrated the critical role of precise process control in flow microreactors for successfully generating and reacting highly unstable organolithium intermediates, specifically dichloromethyl lithium (DCM-Li) and aryl lithium species, which are prone to side reactions in batch systems. Utilizing a continuous flow setup featuring PTFE T-mixers (ID 0.5 mm) and PFA tubular reactors (ID 0.8 mm), the authors achieved controlled residence times of 0.5 to 1.0 s (0.5 s for metalation reaction and 1.0 s for quench reaction) at −30 °C (Figure 17A). Selective monoaddition is proposed to proceed through tetrahedral intermediates, which are putative and stabilized. This flow method provided efficient access to a wide range of α,α′-bis-chloroketones **27** (approach A) and aryl keto acetals **28** (approach B) with high throughputs. Fabio Lima et al. [47] also developed a continuous flow process for synthesizing heteroaryl sulfinates **29** via bromine–lithium exchange and subsequent quenching with sulfur dioxide (Figure 17B). Utilizing a microreactor system comprising PTFE T-mixers (ID 0.5 mm) and PFA tubular reactors (ID 0.8 mm), the authors achieved short residence times of ≤1 s, which were critical for maintaining the stability of reactive aryllithium species. This precise process control in flow enabled the generation and immediate trapping of intermediates to prepare sulfinates in moderate to high yields using 0.4 M aryl bromide in THF, 1.6 M *n*-BuLi in hexane, and 0.5 M SO_2_ in THF. Lithium sulfinate salts synthesized via this flow approach acted as efficient coupling components in Pd-catalyzed cross-coupling, enabling the synthesis of medicinally relevant bis-heteroaryl motifs. Aiichiro Nagaki et al. [48] developed a continuous flow system (Figure 17C) enabling the direct and highly selective synthesis of functionalized ketones **30** from acid chlorides and organolithiums—a transformation traditionally plagued by side reactions in batch reactors. By employing a T-shaped micromixer (ID 250 μm) and microtube reactors and increasing the total flow rate, the authors achieved extremely fast 1:1 micromixing, which was critical for suppressing competitive consecutive and parallel reactions. This flow approach allowed the chemoselective coupling of acid chlorides bearing electrophilic functional groups (e.g., nitro, cyano) with functionalized organolithiums generated in situ via halogen–lithium exchange, producing doubly functionalized ketones in good yields. The method was successfully applied to the synthesis of a key intermediate for GW678248, an HIV nonnucleoside reverse transcriptase inhibitor, with a yield of 52%. Andreas Hafner et al. [49] demonstrated a continuous flow platform enabling the synthesis of diverse boronic acids **31** via organolithium intermediates within a total reaction time of 1 s (Figure 17D). By employing a system comprising a PTFE T-mixer (ID 0.5 mm) and PFA tubular reactors (ID 0.8 mm), the authors achieved efficient halogen–lithium exchange and subsequent electrophilic borylation. Crucially, they optimized the process by correlating yield with total flow rate rather than residence time alone, discovering that increasing the total flow rate to >14 mL/min significantly enhanced mixing efficiency and conversion. This flow approach effectively converted both electron-rich and electron-poor substrates into the corresponding boronic acids in good to excellent yields. Operating at a total flow rate of 20.5 mL/min, it achieved a throughput of 5.1 mmol/min, striking an optimal balance between throughput, material consumption, and pump limitations. The flow platform’s versatility was further extended to the multigram synthesis of various alcohols and aldehydes, achieving high yields of 92% to 97%. Aiichiro Nagaki et al. [50] demonstrated effective control over the reaction process using a flow microreactor system, achieving highly chemoselective reactions between functionalized aryllithium compounds and difunctional electrophiles with short residence time. By employing a continuous flow system with V/T-shaped micromixers (ID 250 μm) and microtube reactors operating at high total flow rates, the authors leveraged extremely fast micromixing to overcome the inherent selectivity challenges of traditional batch methods. This study investigated both the reactions of various electrophilic functionalized aryllithium compounds and the competition between the isocyanate group and other electrophilic groups. Furthermore, the integrated flow system facilitated chemoselective three-component coupling (Figure 17E), synthesizing complex polyfunctional molecules **32** in a high yield (61%) with a productivity of 156 mg/min. This approach serves as an effective protecting-group-free strategy employing organolithium chemistry, while also paving the way for the synthesis of complex polyfunctional organic molecules.

**Figure 18:**
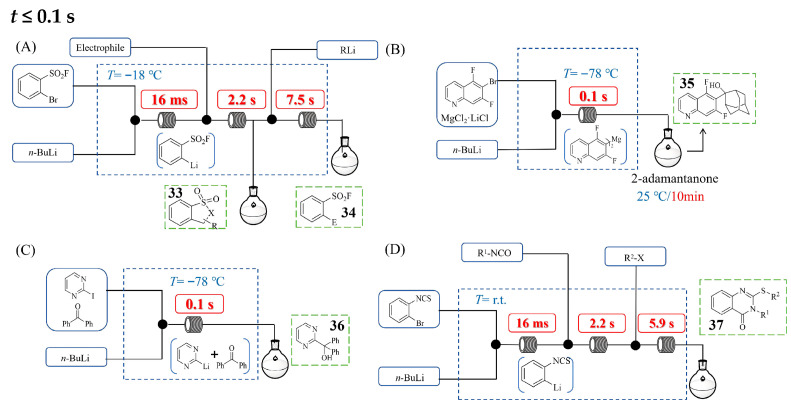
Continuous flow process for organolithium reactions with minimum residence time ≤ 0.1 s. (**A**) The synthesis of *o*-functionalized benzenesulfonyl fluorides and subsequent SuFEx connections. (**B**) Br/Li exchange with in situ trapping: synthesis from 6-bromo-5,7-difluoroquinoline and adamantanone. (**C**) Halogen–lithium exchange under Barbier conditions and in situ trapping with electrophile. (**D**) Integrated process for the synthesis of thioquinazolinones.

Figure 18 presents a continuous flow system for executing organolithium reactions with an ultrashort minimum residence time (≤0.1 s), a regime essential for suppressing the side reactions of transient or unstable organolithium species. Ji-Ho Kang and Dong-Pyo Kim leveraged a capillary microreactor system to achieve the ultrafast synthesis of functionalized benzenesulfonyl fluorides [51]. By meticulously optimizing residence time down to 0.016 s at −18 °C, they successfully generated and trapped highly unstable aryllithium intermediates that rapidly decompose under traditional batch conditions (Figure 18A). This millisecond-scale control suppressed side reactions and enabled efficient functionalization with a range of electrophiles, affording diverse ortho-functionalized sulfonyl fluorides which were further cyclized via intramolecular SuFEx to give products **33** in 27–94% yields. This precise control allowed the reaction involving even unstable organolithium nucleophiles to be directly integrated via SuFEx in a single flow, yielding complex sulfones **34** in 42–72% yields within 10 s. The strategic method developed herein utilizes simple sulfonyl fluoride substrates and the precise control of flow reactors to allow efficient molecular assembly, providing a rapid route to synthesize complex functionalized sulfonyl fluorides. Rodolfo Hideki Vicente Nishimura et al. [52] developed a continuous flow process for generating and trapping unstable heteroaryllithium intermediates to prepare functionalized heterocycles and a typical example for the synthesis of 2-(5,7-Difluoroquinolin-6-yl)adamantan-2-ol **35** in a high yield of 95% is presented in Figure 18B. Utilizing a commercial flow setup with PTFE/PFA T-mixers (ID 0.5 mm) and tubular reactors (ID 0.25/0.8 mm), the authors achieved precise control over residence time, which was critical for managing rapid Br–Li exchange and subsequent transmetalation to the more stable diheteroarylmagnesium species. By maintaining ultrashort residence time between 0.1 and 2.5 s at temperatures ranging from −78 °C to 0 °C, the system effectively suppressed the side reaction pathways that plague these highly reactive organometallics in batch systems. The diheteroarylmagnesium species, successfully prepared under flow conditions, could subsequently be trapped with a wide range of electrophiles, including ketones, aldehydes, allylic bromides, and disulfides. Niels Weidmann et al. [53] demonstrated the critical role of precise residence time control for challenging organolithium-mediated reactions under Barbier conditions, as exemplified by the 99% yield synthesis of diphenyl(pyrimidin-2-yl)methanol **36** shown in Figure 18C. Utilizing a commercial continuous flow setup comprising T-mixers (ID 0.5 mm) and tubular reactors (ID 0.25/0.8 mm), the authors achieved ultrashort, finely tuned residence times ranging from 0.1 to 7.5 s at temperatures between −78 °C and 0 °C. This exact temporal management was essential for the generation and instantaneous trapping of highly reactive organolithium intermediates with various electrophiles, such as aldehydes, ketones, Weinreb amides, and imines, effectively suppressing the side reaction pathways that plague batch systems. The process facilitated the synthesis of diverse functionalized (hetero)arenes with high efficiency, yielding products in good yields (e.g., 99% yield for diphenyl(pyrimidin-2-yl)methanol), even for substrates bearing sensitive ester groups. Heejin Kim et al. [54] developed a continuous flow system for synthesizing heterocyclic thioquinazolinones **37** through precisely controlled organolithium reactions operating at millisecond timescales (Figure 18D). Their integrated continuous flow system enabled the sequential generation and reaction of two highly unstable organolithium intermediates: first an isothiocyanate-functionalized aryllithium, followed by the corresponding lithium thiolate. This approach achieved unprecedented control, maintaining the initial generation of an aryllithium intermediate at room temperature with a residence time of just 16 ms. The flow system design, which incorporated T-shaped micromixers and microtube reactors, combined with precise flow rate control, enabled extremely fast mixing and exact residence time management. This approach allowed the three-step synthesis of various S-benzylic thioquinazolinone derivatives within 10 s in high yields of 75–98%, starkly contrasting conventional methods requiring hours under harsh conditions. The method’s scalability was demonstrated through gram-scale synthesis, producing 1.25 g of a multifunctionalized thioquinazolinone product (91% yield) in 5 min.

### 3.3. Phase Regime

Although most organolithium reactions involve a homogeneous liquid phase, flow chemistry’s ability to handle gas–liquid reactions is also a key advantage, which are difficult in scaled-up batch reactors due to mass transfer limitations. By ensuring highly efficient gas–liquid mixing and mass transfer within specialized reactor designs, flow chemistry enables reproducible and scalable reactions using gaseous reagents in organolithium processes, transforming the previously challenging multiphase syntheses into feasible and efficient processes.

**Figure 19:**
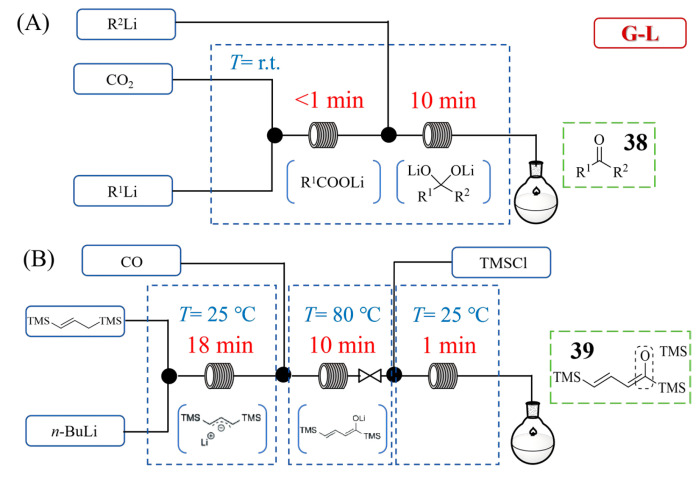
Continuous flow process for multiphase organolithium-mediated reactions. (**A**) Ketone synthesis from organolithiums and CO_2_. (**B**) Carbonylation of 1-silyl-substituted organolithiums under CO pressure.

Jie Wu et al. [55] developed a continuous flow method for synthesizing ketones **38** from CO_2_ and organolithium or Grignard reagents (Figure 19A), demonstrating a significant application of flow chemistry in handling gas–liquid multiphase fast reactions. Their system utilized a precisely controlled flow setup featuring mass flow controllers to meter CO_2_ gas and micromixers to achieve exceptional gas–liquid interfacial contact, realizing enhanced mass transfer that enabled the efficient carboxylation of organometallic reagents. A key discovery was the unprecedented solvent-dependence of organolithium reactivity, where organolithium reagents were less reactive in THF compared with in Et_2_O. To address distinct CO_2_ utilization scenarios, the authors developed two complementary continuous flow systems. For stoichiometric CO_2_, a system without an in-line degasser was designed; for excess CO_2_, another system incorporated an in-line vacuum degasser to remove surplus CO_2_ between reaction steps. The two systems operated efficiently at ambient temperature and pressure to synthesize diverse ketones. This approach not only provides a robust platform for gas–liquid organometallic chemistry but also facilitates a telescoped three-step-one-flow process for the synthesis of ketones in high yields of 72~84%. Takahide Fukuyama et al. [56] developed a continuous flow system that significantly enhanced the gas–liquid process for the carbonylation of sensitive 1-silyl-substituted organolithiums under pressurized CO conditions, for which Figure 19B displays a typical example of a three-consecutive-flow reaction system for the substrate of 1,3-bis(trimethylsilyl)propene to synthesize ketone **39**. Their approach utilized serially connected micromixers, microreactors, and a back-pressure regulator to create pressurized conditions that significantly accelerated the reaction kinetics compared with atmospheric batch processes, in which the microreactor’s miniature channels enabled exceptional gas–liquid interfacial contact through efficient plug flow formation. This intensified continuous flow process reduced CO trapping reaction times from 1 to 10 h in batch to just 4–10 min. Through constructing a three-consecutive-flow reaction system, generation, carbonylation, and reaction with electrophiles of 1-silyl-substituted organolithiums was carried out to deliver the desired products yield of 77~93%.

### 3.4. Synthesis Steps

Organolithium reactions, due to their high reactivity and sensitivity to moisture and oxygen, present significant challenges in traditional batch multi-step syntheses. These challenges are particularly evident in the difficulties associated with the separation and purification of intermediates and compatibility issues with unstable functional groups, which to some extent constrain the overall design efficiency of synthetic routes. Flow chemistry technology, leveraging its modularly integrated continuous flow reactor systems, provides an effective solution for multi-step organolithium reactions. This technology not only significantly enhances synthetic efficiency but also promotes the development of green and scalable synthesis processes.

**Figure 20:**
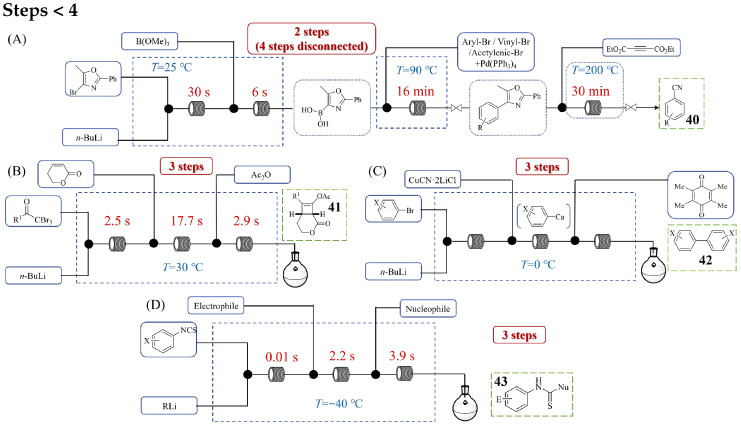
Continuous flow process for multi-step synthesis (steps < 4). (**A**) Cyanide-free cyanation of sp^2^/sp-carbon atoms using an OxBA-based masked cyanide source. (**B**) The synthesis of cyclobutenes via lithium ynolates. (**C**) The generation of organocuprates and biaryl coupling sequence. (**D**) Three-step integrated process for the reaction of the meta- and para-substituted phenyl isothiocyanate with organolithium.

Figure 20 illustrates the continuous flow process for multi-step synthesis involving fewer than four sequential reactions. Brijesh M. Sharma et al. [57] developed a four-step flow method for the cyanide-free cyanation of sp^2^/sp carbons using an oxazole-based masked cyanide source (Figure 20A). Their methodology sequentially combined lithiation–borylation to generate a stable OxBA ((5-methyl-2-phenyloxazol-4-yl) boronic acid) reagent, Suzuki–Miyaura cross-coupling with various aryl/vinyl/acetylenic halides, and a final thermal [4+2]/retro-[4+2] demasking sequence to deliver functionalized nitriles **40**. This orchestrated flow system allowed for the precise and room temperature lithiation/borylation of bromo-oxazole, achieving a 75% yield of the OxBA reagent in a short residence time of 36 s, a substantial improvement over the 90 min needed in batch methods. Subsequent Suzuki–Miyaura cross-coupling, followed by demasking via a [4+2]/retro-[4+2] sequence, afforded the desired benzonitriles in 62–83% overall yield with a total residence time of only 46 min. A unique feature of this flow-based cyanation protocol is its ability to convert a variety of sp^2^ and sp-hybridized carbons into a broad spectrum of aryl nitriles. Aki Kohyama et al. [58] developed a continuous flow synthesis method of functionalized cyclobutenes **41** through a three-step, one-flow process that integrates the generation of highly reactive lithium ynolates and their subsequent [2+2] cycloaddition with α,β-unsaturated esters and the final acetylation of the resulting lithium enolates (Figure 20B). This methodology successfully consolidates multiple transformations—traditionally requiring separate batch operations with cryogenic conditions and laborious intermediate handling—into a single streamlined flow system operating at a practical temperature of 30 °C. The process begins with the flash generation of lithium ynolates via lithium–halogen exchange between α,α,α-tribromomethyl ketones and *n*-BuLi in 2.5 s, followed by immediate cycloaddition (17.7 s) and trapping (2.9 s). By precisely controlling residence times and utilizing in-line Raman spectroscopy for optimization, the system prevents the decomposition of unstable intermediates, enabling the synthesis of various cyclobutene derivatives in good yields. This work demonstrates flow chemistry’s ability to integrate multi-step organolithium transformations into a continuous, scalable process without intermediate isolation, significantly enhancing efficiency and practicality. Kazuhiro Okamoto et al. [59] developed a continuous flow system for the synthesis of functionalized biaryls **42** via organocuprate intermediates (Figure 20C), demonstrating the effective application of flow chemistry in multi-step organolithium processes. The process involves three integrated steps: first, the generation of short-lived aryllithium species from functionalized aryl bromides using *n*-BuLi at 0 °C in a microreactor with precise residence time control; second, transmetalation to organocuprates by mixing with a copper cyanide–lithium chloride complex; and third, oxidative coupling using duroquinone as an oxidant to form biaryl products. The authors optimized chemoselectivity by adjusting the Cu/Li ratio and oxidant choice. Key contributions in this work include achieving high yields of **42** with broad functional group tolerance (e.g., fluoro, bromo, cyano, nitro substituents), overcoming the limitations of traditional palladium-catalyzed methods. Hyune-Jea Lee et al. [60] developed a continuous flow method enabling the multi-step synthesis of functionalized isothiocyanate (NCS)-containing molecules through sequential organolithium reactions, overcoming the inherent instability of meta- and para-NCS-substituted aryllithiums. Their integrated microreactor system facilitated a selective halogen–lithium exchange on iodophenyl isothiocyanates at −40 °C with an ultrashort residence time of 0.014 s, preventing nucleophilic addition to the sensitive NCS group. This precise control allowed the subsequent reaction of the generated aryllithium intermediates with diverse electrophiles (e.g., trimethylsilyl triflate, methyl chloroformate, methyl triflate) within a single flow sequence, achieving high yields (63–97%) of NCS-functionalized products. Furthermore, the platform enabled a three-step integrated synthesis method (Figure 20D) where the NCS group of these products underwent a nucleophilic addition with organolithiums, producing various thioamides **43** in yields of 73~95% within 6.11 s total reaction time. In addition, a hybrid strategy, combining the flow reaction with a flask reaction, enabled the high-yield synthesis of biologically active compounds.

**Figure 21:**
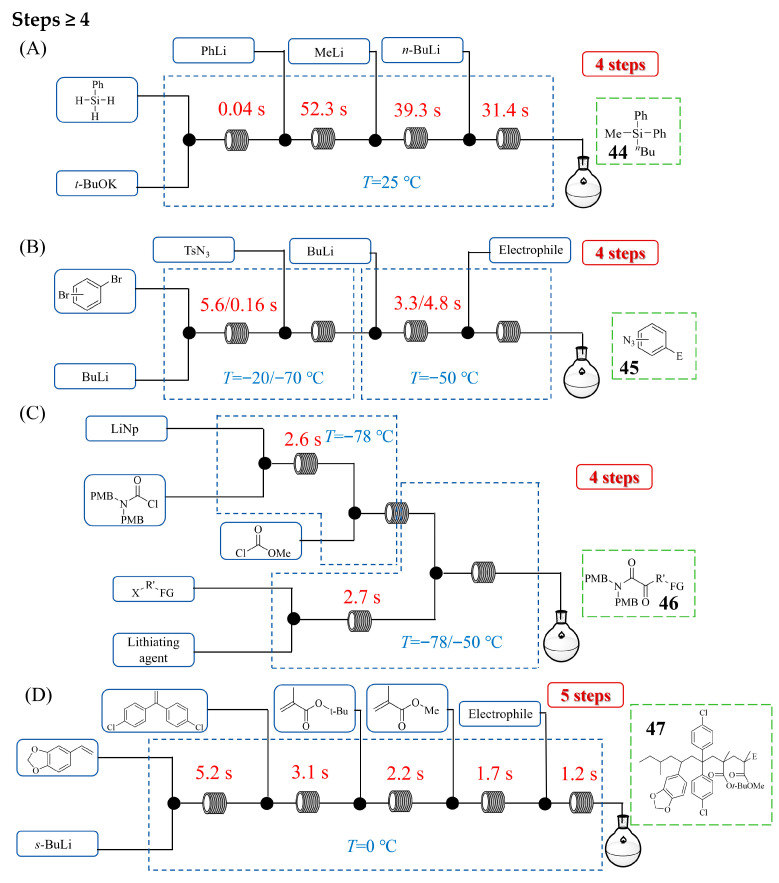
Continuous flow process for multi-step synthesis (steps ≥ 4). (**A**) The combinatorial synthesis of tri-substituted silanes. (**B**) The synthesis of functionalized aryl azides from dibromoarenes. (**C**) Three-component coupling of carbamoyllithium species, methyl chloroformate, and functionalized organolithium reagents. (**D**) Six-component coupling sequence.

Figure 21 presents a continuous flow system designed to efficiently accomplish more complex synthetic sequences comprising four or more steps. Hyune-Jea Lee et al. [61] developed a continuous flow method for the atom-economic, sequential mono-, di-, and tri-functionalization of unactivated hydrosilanes via organolithium reactions catalyzed by Earth-abundant potassium *tert*-butoxide (*t*-BuOK). Figure 21A shows the continuous flow process for the combinatorial synthesis of tri-substituted silanes **44** based on the sequential functionalization of three Si–H bonds of phenylsilane. Their integrated microreactor system enabled the in situ generation of various functionalized organolithiums and their subsequent rapid reaction with hydrosilanes, completing mono-functionalizations within 1 min at room temperature—conditions unattainable using the batch method. The catalytic *t*-BuOK was crucial for accelerating the reaction of silylation, forming a pentacoordinated silicon intermediate that reacted efficiently with organolithiums. This precise temporal control in flow prevented undesirable side reactions, allowing for the sequential addition of different organolithiums (e.g., PhLi, *n*-BuLi, MeLi) to hydrosilanes. This capability facilitated the rapid synthesis (within 3 min) of a diverse library of tetra-substituted silanes in high yields. Daisuke Ichinari et al. [62] developed a continuous flow method enabling the multi-step synthesis of functionalized aryl azides through the generation and reaction of organolithium species bearing a masked triazene group. Their approach overcame the inherent instability of azide groups under traditional organolithium conditions by utilizing a tosyl triazene as a stable synthetic equivalent, which could be subsequently hydrolyzed and removed. Figure 21B shows the continuous flow process for the synthesis of functionalized aryl azides **45** from dibromoarenes. The integrated flow system facilitated a precise, sequential process: a selective bromine–lithium exchange on polybromoarenes, immediate trapping with tosyl azide to install the masked azide, and a subsequent second lithiation exchange on the remaining bromide to introduce various electrophiles. This orchestrated sequence, with an effectively controlled residence time and reaction temperature, successfully produced a diverse range of functionalized aryl azides in high yields of 43~80%. In addition, the continuous flow process was further applied to synthesize a precursor for protein tyrosine phosphatase inhibitors and a P2Y_14_ receptor antagonist, demonstrating the power of flow reactors in executing complex, multi-step organolithium transformations. Aiichiro Nagaki et al. [63] demonstrated a multi-step continuous flow synthesis of functionalized α-ketoamides via the generation and reaction of highly unstable carbamoyllithium species, a transformation that is impractical in batch reactors due to rapid decomposition. Their integrated microreactor system enabled a precise three-component coupling sequence: the initial reductive lithiation of a carbamoyl chloride, immediate trapping with methyl chloroformate, and subsequent reaction with a functionalized organolithium reagent. Figure 21C shows the continuous flow system for the three-component coupling of a carbamoyllithium species, methyl chloroformate, and functionalized organolithium reagents to synthesize functionalized α-ketoamides **46**. This flow process successfully produced a diverse range of α-ketoamides in good yields of 51~70%, a significant improvement over low-yielding batch attempts. The methodology was successfully applied to a formal synthesis of GW356194, a potential sodium channel blocker, showing its utility for constructing complex molecules through sequential organolithium reactions. Kazuhiro Okamoto et al. [64] developed a continuous flow method enabling multi-step, sequence-defined synthesis using organolithium chemistry, achieving up to six-component reactions in a single integrated process without intermediate purification (Figure 21D). Their system utilized precise flow control to execute a sequential monoaddition strategy, where an organolithium initiator first reacts with functionalized styrenes, then with diarylethylenes, followed by various methacrylates, and is finally trapped by an electrophile. This approach overcame the traditional limitation of uncontrolled polymerization, allowing the selective formation of monoaddition products at each step. By leveraging efficient mixing and rapid reactions in serially connected microreactors, the authors suppressed side reactions and achieved a high yield of **47** (76% for the full six-component sequence) with a total residence time of only 13.4 s. This work establishes flow chemistry as a powerful platform for complex multi-step organolithium reactions, providing a robust and efficient route to structurally diverse, sequence-controlled oligomers.

### 3.5. Process Safety

Flow chemistry offers an inherently safer approach for high-risk organolithium processes by addressing the key limitations of batch reactors. Through engineered continuous flow design, it effectively mitigates hazards such as thermal runaway, localized overheating, and operator exposure to sensitive reagents. Organolithium compounds are not only highly sensitive to moisture and oxygen but also generate intense heat upon reaction—risks exacerbated in batch systems by poor mixing and slow heat transfer. In contrast, flow systems enable instantaneous heat dissipation and operate with minimal reactor holdup, physically restricting hazardous energy and materials to a small, well-controlled volume. The inherent safety characteristics of flow chemistry lowers the operational risk and provides a safer, more scalable platform from research to industrial production.

**Figure 22:**
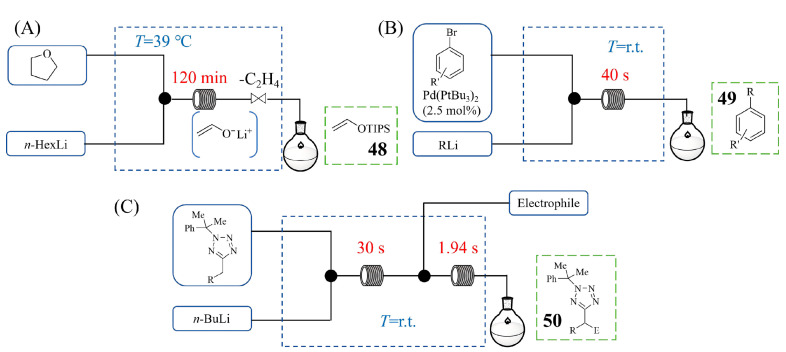
Continuous flow process for the safe handling of organolithium-based reactions. (**A**) Generation of lithium ethenolate by lithiation of tetrahydrofuran. (**B**) Pd-catalyzed cross-coupling reactions. (**C**) Lithiation–substitution of 5-alkyltetrazoles.

Parth Naik et al. [65] developed a continuous flow process for the safe generation and utilization of lithium ethenolate, a highly reactive intermediate traditionally accessed via the hazardous lithiation of tetrahydrofuran. Recognizing the significant safety concerns associated with a conventional batch process—which involves the exothermic generation of flammable gas (e.g., ethylene)—the authors translated this transformation into a continuous flow system, where lithium ethenolate is captured by TIPSOTf (triisopropylsilyl triflate) for the formation of TIPS-enol ether **48** (Figure 22A). This approach provided superior control over the reaction’s exotherm and enabled the safe, controlled release of gaseous by-products. By employing a design of experiments (DoE) approach, they optimized the flow conditions (39 °C, 120 min) using *n*-HexLi for the synthesis of **48**, a safer alternative to *n*-BuLi that produces hexane instead of gaseous butane. The corresponding silylenol ether is a versatile building block, whose application in various cycloaddition reactions has been investigated. This flow strategy demonstrated operational stability over several hours, facilitating a gram-scale synthesis of the silylenol ether product. Jacopo Brucoli et al. [66] developed a continuous flow process for Pd-catalyzed cross-coupling reactions of organolithium reagents with aryl bromides that enable safe operation under aerobic conditions (Figure 22B). Their flow system safely managed the high reactivity and air/moisture sensitivity of organolithiums by utilizing short residence times (40 s) within a small reactor volume (0.389 mL), which enables the facile and safe scale-up for large-scale cross-coupling reactions with organolithium compounds. Notable for its operational simplicity, requiring no pre-catalyst activation or inert atmosphere, this method also exhibits a broad substrate scope, providing cross-coupling products **49** in moderate-to-good yields with high productivity. Jeff Y. F. Wong et al. [67] developed a continuous flow process for the synthesis of **50** through the heterobenzylic C-H functionalization of 5-alkyltetrazoles that specifically addressed significant process safety concerns associated with the exothermic lithiation step (Figure 22C). Their key safety advancement was the use of thermal imaging to identify and quantify a dangerous exotherm that caused the ethereal solvent (THF) to boil within the reactor under initial flow conditions. This diagnostic approach prompted a critical solvent switch to a higher boiling temperature toluene/TMEDA mixture, which eliminated the boiling hazard while maintaining reactivity. The optimized flow system enabled precise control over the highly exothermic metalation, allowing it to be conducted safely at room temperature with an extremely short residence time (e.g., 1.94 s for electrophilic trapping step). This controlled environment enabled the effective usage of unstable organolithium intermediates and achieved a high productivity rate of 141 g·h^−1^, demonstrating flow chemistry’s unique capability to safely manage hazardous exotherms that are challenging to control in batch reactors.

**Figure 23:**
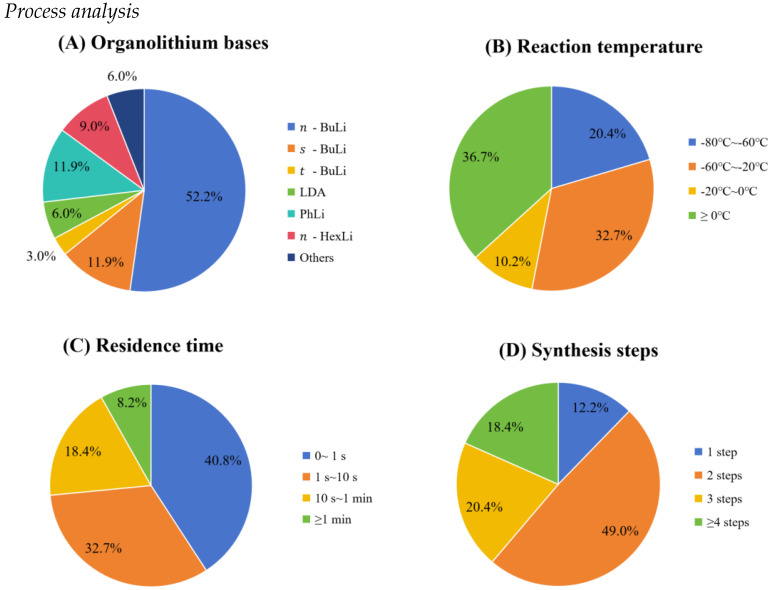
Analysis of the literature on continuous flow organolithium reactions.

The development of continuous flow processes for organolithium-based synthesis typically starts with a comprehensive literature review and initial small-scale batch experiments. These preliminary studies are crucial for elucidating the fundamental characteristics of the target reaction, including its kinetics, thermodynamics, and potential exothermic risks. Based on these findings, the key process parameters and reagents selection for continuous flow development (e.g., reaction temperature, residence time, and organolithium base) have been identified and their appropriate experimental ranges can be defined. A systematic analysis of 49 published articles (discussed in Section 2 and Section 3) was conducted to discuss the key parameters utilized in organolithium-based reactions (Figure 23). This analysis specifically examines the application of flow chemistry in organolithium reactions from several critical perspectives: the selection and use of organolithium bases, adopted reaction temperature windows, characteristic residence times, and the number of integrated synthesis steps. The compiled data reveals prevailing trends, such as the predominance of short residence times and the potential for multi-step one-flow sequences, which represents a capability that is challenging to achieve in traditional batch setups.

Figure 23A shows the utilization of various organolithium bases in continuous flow applications. As illustrated in the figure, *n*-BuLi is the most extensively employed reagent, accounting for a significant majority (52.2%) of the reported cases. This prevalence can be attributed to its strong base strength, good nucleophilicity, wide commercial availability, and well-understood reactivity. The *sec*-butyllithium (*s*-BuLi) and *tert*-butyllithium (*t*-BuLi) isomers represent 11.9% and 3.0% of usage; their application is often driven by the need for greater base strength and/or altered selectivity, despite their higher cost and handling challenges. The LDA is utilized in 6.0% of cases and is typically selected for its high basicity and non-nucleophilic character in regioselective deprotonations. Other reagents, such as phenyllithium (PhLi, 11.9%) and *n*-hexyllithium (*n*-HexLi, 9.0%), find application by virtue of their distinctive reactivity and are commonly used for specific transformations. The distribution shown in the figure highlights a practical preference for cost-effective and versatile reagents, such as *n*-BuLi. In contrast, other organolithium bases are typically chosen for more targeted applications that demand unique selectivity or reactivity. Figure 23B displays the distribution of the minimum required reaction temperature for continuous flow organolithium reactions, offering a direct view of their practical temperature operating windows. The largest single segment of reactions (36.7%) in the figure proceeds efficiently at or above 0 °C, highlighting a key advantage of continuous flow technology: the enhanced heat transfer and precise temperature control in microreactors enable the safe handling of these highly exothermic processes at more accessible and energy-efficient temperatures. The relatively small percentage (10.2%) of reactions in the −20 °C to 0 °C range also demonstrates the practicality of flow systems, as this temperature range is both accessible and well controlled in lab or industrial environments. A substantial portion of reactions (32.7%) operates in the very low temperature range of −60 °C to −20 °C, indicating that while flow systems facilitate the rise in operation temperature, many transformations still require controlled adequately low temperatures to suppress side reactions and ensure selectivity. Notably, a considerable minority of reactions (20.4%) necessitate an ultra-low temperature (−80 °C to −60 °C), underscoring the continued need for specialized cooling capabilities even in flow systems, albeit with improved safety and control compared with traditional batch reactors. The temperature distribution in the figure shows that flow chemistry does not completely eliminate the need for low temperatures but dramatically broadens the viable temperature window for conducting organolithium reactions safely and reproducibly. The distribution of the minimum required residence time for organolithium reactions (Figure 23C) reveals a striking dominance of extremely fast kinetics. As shown, a remarkable 40.8% of reactions complete within 0~1 s, forming the largest segment, followed closely by the 1 s~10 s range (32.7%). This means that nearly three-quarters (73.5%) of all organolithium transformations finish in under 10 s. A smaller but significant proportion (18.4%) necessitates a slightly longer timeframe of 10 s to 1 min, while only 8.2% of the discussed cases require 1 min or more. The distribution in the figure powerfully indicates the exceptionally high reactivity of organolithium species. The prevalence of sub-10-s timescales highlights a critical advantage of continuous flow technology, its ability to provide precise residence time control and highly efficient mixing/heat transfer, which is essential for managing such rapid exothermic processes that would be difficult to control effectively in traditional batch reactors. The distribution of synthesis steps for organolithium reactions (Figure 23D) reveals a clear preference for streamlined processes in continuous flow systems. As illustrated, two-step reactions constitute the largest segment (49.0%), showing their prevalence as an optimal strategy for common sequences such as lithiation followed by electrophilic quenching or tandem reactions that integrate a second transformation. In contrast, single-step reactions represent a small proportion (12.2%), which may reflect the relative simplicity of such transformations or their limited application scope in complex synthesis. Notably, multi-step sequences (three steps: 20.4%; four or more steps: 18.4%) collectively account for a significant share (38.8%), highlighting the capability of continuous flow platforms to efficiently perform complex, multi-step synthetic routes—a task that is often challenging in batch reactors due to highly reactive organolithium intermediate handling and accumulation. This distribution shows the versatility of flow chemistry in accommodating both straightforward lithiation-trapping protocols and complex multi-step cascades, with two-step processes emerging as the most favored balance between synthetic utility and operational efficiency. In addition, an analysis of the literature cases indicates that approximately 90% of continuous flow organolithium reactions are conducted under homogeneous liquid phase conditions. This predominance is attributed to the excellent solubility of both the organolithium reagents and their substrates in common aprotic organic solvents (e.g., THF, Et_2_O, or hydrocarbons), which facilitates efficient mixing and rapid reaction kinetics. In contrast, the minority (about 10%) of systems involve gas–liquid biphasic regimes. These typically arise when gaseous reactants such as carbon dioxide (for carboxylation) are directly introduced into the reaction stream. The transition from a homogeneous to a biphasic system presents distinct engineering considerations, particularly in terms of mass transfer and reactor design, to ensure efficient contact between the phases. The analysis above serves as a reference for researchers in the development of continuous flow organolithium methodologies.

## 4. Flow Chemistry Process Research in Organolithium Reactions

**Figure 24:**
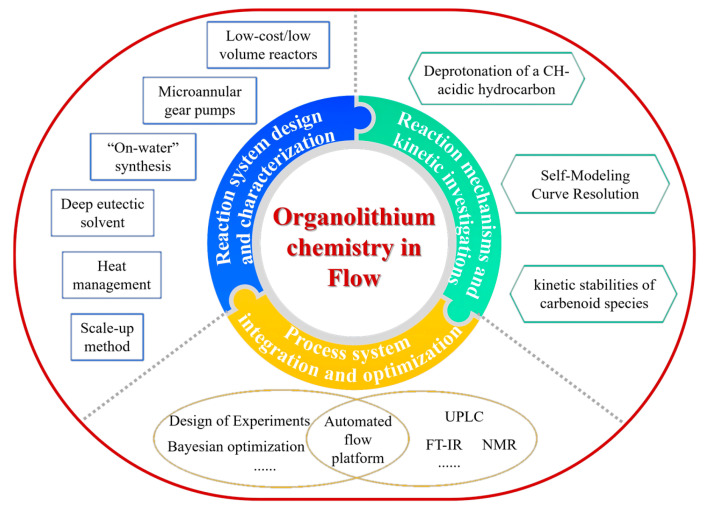
Process research in continuous flow organolithium reactions.

Flow chemistry has improved the application of organolithium reactions by enabling the precise control of reaction parameters, enhancing operational safety, and facilitating scalability through innovative reactor designs. From a process engineering perspective, recent relevant process studies have primarily centered on three aspects: reaction system design and characterization, reaction mechanistic and kinetic investigations, and integrated process system and optimization (Figure 24). The following subsections will elaborate on each of these aspects in detail.

### 4.1. Reaction System Design and Characterization

In the development of synthesis processes for high-value-added chemicals, especially pharmaceuticals, substrate availability is often limited. This constraint necessitates the adoption of efficient approaches that minimize material consumption. Traditional flow reactors for organolithium chemistry require high flow rates to ensure efficient mixing, leading to substantial material demands. James A. K. Cochrane et al. [68] developed low-cost, low-volume reactors for aryllithium flash flow chemistry, utilizing commercially available mixers like chip-based and tee-piece designs to operate at flow rates below 5 mL/min. Their work reduced substrate requirements to as little as 100–300 mg by characterizing mixer performance through model lithium–halogen exchange reactions, enabling efficient small-scale synthesis for pharmaceutical chemistry applications. Julien Haber et al. [69] employed micro annular gear pumps as active mixers to minimize material consumption in organometallic reactions, achieving mixing times under 50 ms even at flow rates less than 1 mL/min and demonstrating robustness against clogging for over 2 h through controlled precipitation tests. This approach allowed for a 10–20 fold reduction in material use while maintaining high yields by optimizing hydrodynamic parameters.

Organometallic reactions typically require anhydrous, cryogenic conditions and an inert atmosphere, entailing a high energy consumption and operational complexity. Florian F. Mulks et al. [70] pioneered continuous organometallic reactions in deep eutectic solvents (DESs), such as glyceline (choline chloride/glycerol) and reline (choline chloride/urea), at room temperature. Employing a segmented flow regime, they obtained high yields without clogging, as the DES phase dissolved lithium salts and exhibited good moisture tolerance. This system allowed for stable operation with organolithium and Grignard reagents, demonstrating the potential for sustainable and energy-efficient processes by eliminating cryogenic conditions. “On-water” reactions offer an eco-friendly alternative to organic solvents. Jacopo Brucoli et al. [71] designed an ultrafast continuous flow method for the “on-water” addition of organolithium reagents to imines in a continuous stirred tank reactor (CSTR), producing 5 g of amine in 3 min for the reaction with fast reaction times (10–20 s) with a single 2.5 mL CSTR. A calorimetric study revealed the exothermic nature of the reactions, and a telescoped process including synthesis, in-line extraction, and separation was developed to demonstrate the feasibility of scaling-up such high-risk reactions safely and productively.

Flow chemistry provides distinct advantages for investigating the thermal characteristics of organolithium reactions, as it enables precise control over mixing efficiency and heat dissipation, thereby facilitating the safe and accurate determination of reaction enthalpies for highly exothermic and fast transformations. This is particularly crucial for process development and scale-up, where traditional batch calorimetry often struggles with slow heat transfer, potential side reactions, and unstable intermediates, leading to unreliable thermochemical data. Frederik Mortzfeld et al. [72] pioneered a meso-flow calorimeter utilizing a jacketed tube reactor with integrated temperature sensors and DynoChem software to deconvolute reaction enthalpy from spatial temperature profiles. They validated this approach with the benchmark reaction of ethanol and hexyllithium, achieving a Δ*H* of −198 kJ/mol, which aligned closely with batch calorimetry results (−206 kJ/mol), demonstrating a mere 4% deviation and underscoring the method’s accuracy. Subsequently, the system was applied to the lithiation of 3-chlorobromobenzene, yielding a Δ*H* of −93 kJ/mol, and the data successfully supported pilot plant scale-up by defining cooling requirements and safety parameters. Gang Fu et al. [73] employed a modular 3D-printed isothermal continuous flow calorimeter that directly measures heat flux via Seebeck elements, allowing for the high-resolution thermal analysis of flash chemistry. In the reaction of hexyllithium with ethanol, an average Δ*H* of −297.6 kJ/mol was obtained for different solvent compositions, and the avoidance of undesired reactions with 2-methyltetrahydrofuran showcased the flow system’s ability to provide clean thermodynamic data. By combining computational fluid dynamics (CFD) simulations of mixing efficiency with a DoE study, they achieved the highly selective synthesis of a *tert*-butyl ester and an alcohol in a microstructured flow calorimeter and successfully deconvoluted their respective reaction enthalpies. The lithium–halogen exchange and its iodine quenching demonstrated that the quenching step was more exothermic than the lithiation, providing critical insights for reaction design and safety assessments. Jeff Y. F. Wong et al. [67] leveraged thermal imaging to identify and manage exotherms in the metalation–substitution of 5-alkyltetrazoles, focusing on process safety and productivity. Thermal imaging revealed significant temperature rises during the reaction period, prompting a switch to toluene/TMEDA solvents to prevent solvent boiling and ensure stable operation. This real-time thermal monitoring enabled the identification of potentially unsafe exotherms for the development of a reliable continuous flow method.

The scale-up from lab-scale microreactors to pilot-scale millireactors involves unpredictable changes in mixing and heat transfer, which pose risks to process safety and product yield and therefore require a deep understanding of their effects on reaction kinetics. Shusaku Asano et al. [74] numerically and experimentally quantified microreactor performance for fast reactions such as organolithium reactions, creating charts based on Damköhler and *β* numbers to assess the impact of mixing and heat transfer on the reaction behavior. Their methodology provided criteria for reactor scale-up and was validated using protecting-group-free halogen–lithium exchange experiments. Verena Fath et al. [75] proposed a model-based approach using in-line FT-IR spectroscopy to predict the transition from micro-scale to milli-scale reactors, incorporating heat and mass balances along with kinetic models derived from lab experiments. Their work emphasized the effects of axial dispersion and hot spot formation, showing that detailed models could accurately predict conversion profiles and temperature rises, thus facilitating safe process scale-up with reduced experimental trials.

### 4.2. Reaction Mechanisms and Kinetic Investigations

Flow chemistry has emerged as a powerful tool for elucidating reaction kinetics and mechanisms in organolithium chemistry. The continuous flow environment ensures well-defined mixing, near-isothermal conditions, and ideal plug flow behavior, which are essential for obtaining reliable kinetic data and probing transient intermediates that are inaccessible in traditional batch reactors.

A prime example is the work by Verena Fath et al. [76], who utilized in-line FT-IR spectroscopy coupled with a microreactor to investigate the deprotonation kinetics between a CH-acidic hydrocarbon and *n*-BuLi. The study revealed complex, broken reaction orders, which were attributed to the postulated mechanism involving aggregation equilibrium of butyllithium species in tetrahydrofuran. Building directly upon this foundational kinetic study, the same research group [75] extended their approach to model-based scale-up predictions. Using the kinetic parameters and mechanistic understanding derived from the microreactor experiments, they developed a comprehensive model incorporating reactor-specific characteristics such as axial dispersion, residence time distribution, and heat transfer coefficients. This model successfully predicted the performance of the deprotonation reaction in larger millireactors, demonstrating a seamless transition from kinetic analysis to practical application. In a related methodological advancement, Verena Fath et al. [77] further optimized kinetic data acquisition by implementing non-steady-state experiments using a decreasing flow rate gradient. This approach, combined with self-modeling curve resolution (SMCR) analysis of the in-line FT-IR spectra, significantly reduced the experimental time and material consumption required for kinetic profiling. The SMCR technique allowed for the nearly calibration-free evaluation of reaction progress, which is particularly beneficial for reactions involving unstable intermediates where traditional calibration methods are challenging. The reliability of this method was validated against steady-state experiments, showing excellent agreement in extracted rate constants while offering a more efficient and resource-effective alternative to kinetic studies of complex organolithium reactions. Kazuhiro Okamoto et al. [78] employed a flash quench-flow microreactor system to study the stability and reactivity of short-lived lithium carbenoid species. By precisely controlling residence times on a subsecond scale, the authors measured the decomposition kinetics of these highly reactive intermediates. Strong correlations were observed between the calculated carbon–halogen bond dissociation energies in lithium carbenoid intermediates and experimental kinetic data. These correlations not only enabled the prediction of reaction rates but also provided reasonable interpretations for ultrashort-lived reactive species. Collectively, these studies illustrate the important impact of flow chemistry on kinetic and mechanistic investigations in organolithium chemistry.

### 4.3. Process System Integration and Optimization

Flow chemistry has changed the handling manner of organolithium reactions, which now necessitates the optimization of these complex and rapid flow processes. The integration of process optimization strategies, such as machine learning algorithms and real-time analytics, offers significant advantages over traditional methods like one-factor-at-a-time (OFAT) or design of experiments (DoE). These approaches reduce experimental effort, enable the rapid acquisition of high-quality data, and facilitate the identification of optimal conditions by efficiently exploring multidimensional parameter spaces, thereby accelerating reaction development and improving sustainability in chemical synthesis.

In the study by Dogancan Karan et al. [79], a machine learning workflow coupled with a flow chemistry platform was developed for the multi-objective optimization of a lithium–halogen exchange reaction. The platform precisely controlled temperature, residence time, and stoichiometry, while employing the Thompson sampling efficient multi-objective optimization (TSEMO) algorithm to balance yield and impurity as conflicting objectives. Through various optimization campaigns, the algorithm effectively identified conditions that captured the optimal trade-off between yield and impurity. The machine learning workflow proved to be a robust and data-efficient strategy that yielded rich information regarding the reaction, outperforming conventional single-objective, OFAT, and DoE methods. Gwang-Noh Ahn et al. [80] presented an automated microreactor platform (AMP) that integrated Bayesian optimization for the autonomous self-optimization of ultrafast thioquinazolinone synthesis via organolithium intermediates. The AMP controlled flow rate, reaction volume, and temperature, utilizing in-line FT-IR for real-time yield monitoring. The AMP demonstrated a robust performance and high efficiency by not only executing 80 pre-planned experiments with precision but also autonomously optimizing reaction conditions using Bayesian optimization. The platform successfully handled complex optimizations involving categorical variables, identifying phenyllithium (PhLi) as the superior choice for synthesizing thioquinazolinone. Moreover, it accomplished the combinatorial synthesis of a nine-membered S-benzylic thioquinazolinone library within 20 min under autonomously determined conditions. Transitioning to detoxification applications, Valmir Baptista da Silva et al. [81] applied a modified Nelder–Mead optimization algorithm in flow to neutralize a sulfur mustard simulant (chloroethyl ethyl sulfide) using PhMgBr or PhLi. The algorithm optimized residence time, stoichiometry, and temperature for full neutralization with PhMgBr in flow conditions. Flow conditions accelerated reaction kinetics by at least 1.3-fold compared with batch conditions, and the process was scaled up to multigram levels, demonstrating practical utility for hazardous compound handling. This work emphasized the algorithm’s efficiency in converging to optimal settings with minimal experimental cost, showcasing flow chemistry’s safety benefits and the value of black box optimization for complex reaction networks. Verena Fath et al. [82] combined in-line FT-IR spectroscopy and online mass spectrometry in a self-optimizing platform for two different reaction types (organolithium reaction and epoxide synthesis). The system employed a modified Simplex algorithm and design of experiments to maximize product yield and purity by monitoring the main product and by-product simultaneously. Through the model-free approach, it reliably identifies optimal conditions for reactions with complex mechanisms and numerous variables, delivering profound process understanding without requiring prior mechanistic knowledge. Beyond process optimization, integrating multiple analytical tools to realize the real-time monitoring of multi-step reactions is essential for achieving Quality by Design (QbD) in pharmaceutical manufacturing. Peter Sagmeister et al. [83] integrated multiple process analytical technology tools, including in-line IR, NMR, and online UPLC, into a modular flow platform for the real-time monitoring of multi-step organolithium reactions. This system enabled rapid data acquisition (17 iterations, <2 h) for optimization, leading to a scaled-out process that produced 4.9 g of target product with 70% yield and a productivity of 4.2 g/h, highlighting the importance of comprehensive reaction process understanding for industrial applications.

## 5. Conclusions

Flow chemistry, leveraging advantages such as efficient mixing, precise mass and heat transfer control, and modular system integration, has successfully enabled the safe and effective regulation of highly reactive, low-temperature-dependent organolithium-based reaction processes. This review analyzes the application of flow chemistry in such reactions from a process viewpoint. A critical examination of organolithium-based reactions from the perspectives of key compound synthesis in the pharmaceutical field, novel methodologies, and fundamental process research consistently shows that continuous flow systems offer unparalleled advantages in addressing their inherent challenges. Despite significant progress, challenges persist, particularly in mitigating practical issues such as solid deposition and reactor clogging and in achieving the fully predictive scale-up of complex organolithium reactions. Future efforts should, therefore, be directed toward addressing these persistent challenges while expanding the exploration of continuous flow systems for a broader range of organolithium-based transformations under sustainable conditions. While initial investment costs for multipurpose flow equipment remain a consideration, increasing commercial availability, modular system designs, and standardized interfaces are progressively enhancing the accessibility and cost-effectiveness of this technology for academic and industrial laboratories alike. Through the collaboration of synthetic chemists and process engineers, organolithium-based reactions performed in flow reactors are poised to play an increasingly important role in synthetic chemistry.

## Data Availability

No new data were created or analyzed in this study. Data sharing is not applicable to this article.
